# Rhizosphere Metagenomics of *Paspalum scrobiculatum* L. (Kodo Millet) Reveals Rhizobiome Multifunctionalities

**DOI:** 10.3390/microorganisms7120608

**Published:** 2019-11-23

**Authors:** Ratna Prabha, Dhananjaya P. Singh, Shailendra Gupta, Vijai Kumar Gupta, Hesham A. El-Enshasy, Mukesh K. Verma

**Affiliations:** 1 Chhattisgarh Swami Vivekananda Technical University, Bhilai, Chhattisgarh 491107, India; ratnasinghbiotech30@gmail.com (R.P.); mkseem670@gmail.com (M.K.V.); 2 ICAR-National Bureau of Agriculturally Important Microorganisms, Indian Council of Agricultural Research, Kushmaur, Maunath Bhanjan 275101, UP, India; 3 Department of Systems Biology and Bioinformatics, University of Rostock, Rostock 18057, Germany; shailendra.gupta@uni-rostock.de; 4 Department of Chemistry and Biotechnology, ERA Chair of Green Chemistry, Tallinn University of Technology, 12618 Tallinn, Estonia; vijaifzd@gmail.com; 5 Institute of Bioproduct Development, Universiti Teknologi Malaysia, Skudai 81310, Johor Bahru, Johor, Malaysia; henshasy@ibd.utm.my

**Keywords:** *Paspalum scrobiculatum*, kodo, millet, rhizosphere, metagenomics, metabolic functions

## Abstract

Multifunctionalities linked with the microbial communities associated with the millet crop rhizosphere has remained unexplored. In this study, we are analyzing microbial communities inhabiting rhizosphere of kodo millet and their associated functions and its impact over plant growth and survival. Metagenomics of *Paspalum scrobiculatum* L.(kodo millet) rhizopshere revealed taxonomic communities with functional capabilities linked to support growth and development of the plants under nutrient-deprived, semi-arid and dry biotic conditions. Among 65 taxonomically diverse phyla identified in the rhizobiome, Actinobacteria were the most abundant followed by the Proteobacteria. Functions identified for different genes/proteins led to revelations that multifunctional rhizobiome performs several metabolic functions including carbon fixation, nitrogen, phosphorus, sulfur, iron and aromatic compound metabolism, stress response, secondary metabolite synthesis and virulence, disease, and defense. Abundance of genes linked with N, P, S, Fe and aromatic compound metabolism and phytohormone synthesis—along with other prominent functions—clearly justifies growth, development, and survival of the plants under nutrient deprived dry environment conditions. The dominance of actinobacteria, the known antibiotic producing communities shows that the kodo rhizobiome possesses metabolic capabilities to defend themselves against biotic stresses. The study opens avenues to revisit multi-functionalities of the crop rhizosphere for establishing link between taxonomic abundance and targeted functions that help plant growth and development in stressed and nutrient deprived soil conditions. It further helps in understanding the role of rhizosphere microbiome in adaptation and survival of plants in harsh abiotic conditions.

## 1. Introduction

Crop plant rhizosphere harbors a huge collection of mutualistic microbial population which encodes metabolic activities supporting the growth and development of the host and associative organisms [1]. Soil bacteria in close propinquity to the plant roots, i.e., the rhizosphere exhibit deep impact on nutrient management and plant defense against biotic and abiotic stresses [2]. Rhizosphere associated microbial communities play a key role in different biogeochemical cycles [3]. A vast microbial majority with interactive functions in the natural habitats still remains uncharacterized due to the limitations of culturability on media conditions [4,5]. This is specifically true in the dynamic biological systems like rhizosphere, which harbors complex microbial diversity and metabolic functions [6,7]. Therefore, for characterizing complex rhizosphere communities and linking functionalities, metagenomics has provided access to the rich pool of genomes in a particular microenvironment [8]. Understanding how different microbial communities in the rhizosphere influence plant performance and productivity using metagenomics can open new avenues for devising eco-friendly ways to cater benefits from microbe-mediated agricultural technologies [9].

*Paspalum scrobiculatum* (kodo or Indian crown grass) is among the ancient grain millets grown in many parts of India, Philippines, Indonesia, Thailand, and West Africa [10], where it is consumed as nourishing healthy and vitality foods in rural areas [11]. As a drought-tolerant and hardy monocot crop especially confined to semi-arid regions, kodo is grown on about 907,800 ha of land annually with the approximate annual production of 310,710 tons [12]. The crop grains possess high-value proteins (11%), carbohydrates (66.6 g per 100 g of grains equivalent to 353 kcal), low fat (3.6 g per 100 g) with iron (25.86 to 39.6 ppm), calcium (27/100 mg) and antioxidant free-radical scavengers [13]. Kodo plants exhibit medicinal attributes like antidiabetic and antirheumatic activities, cures wounds and possesses a tranquilizing effect [12,13]. As against rice and wheat, which contain 0.2% and 1.2% fiber content, kodo is fiber rich (9%) and thus, a beneficial food source for subsistence farming communities in many regions in India and Africa. 

Having said that the plant rhizosphere is dynamic and live ecosystem inhabited by diverse microbial communities with apparent multifunctions [14], focused attention is required to decipher inhabitants of the millet rhizosphere and link communities with the multifunctionalities that favor plant and soil health in such difficult ecological conditions. We analyzed microbial communities and functions in the kodo millet rhizosphere metagenome and identified overall taxonomic abundance of communities, their functional pathways and metabolism in this rhizosphere and establish their role in stress responses, adaptation to abiotic stresses, nutrient recycle, xenobiotic degradation, carbon fixation, plant growth promotion, and disease resistance. The study indicated multi-functions of the microbial communities that help plant growth and development under water deficit drought stress in the rhizosphere. It further extends our understanding on the role of rhizosphere microbiome in adaptation and survival of plants growing under nutrient deprived conditions. 

## 2. Materials and Methods

### 2.1. Soil Sampling and Analysis

*P. scrobiculatum* L. (kodo variety IK 1) was grown as a trial crop for the Rabi season at S.G. College of Agriculture and Research, Jagdalpur, Chhattisgarh, India (19.07N;81.96E). The crop was sown in the last week of February, 2017 in the Experimental Research Farm and was maintained as per the standard agronomic practices [15]. The rhizosphere of two months old plants (6 plants per location; total 5 locations in 4500 sq ft plot; 6–10 cm depth from the top soil) was collected along with the adhering soils, pooled together, refined, and made free from root hairs before processing for metagenomic DNA extraction. Since the ear heads of the crop gains physiological maturity in 100 days (https://www.agrifarming.in/kodo-millet-farming-cultivation-practices; website visited on 17.9.2019), the sampling of the rhizosphere soil was performed prior to the onset of blooming stage in order to analyze the microbial community structure and function before the most critical stage of the crop cycle. The sample was stored in −80 °C until the metagenomic DNA extraction was performed. Analysis of the rhizosphere soil was performed at Center for Analytical Research and Studies, Maharashtra Institute of Technology, India. 

### 2.2. Metagenomic DNA Extraction and Sequencing

Isolation of the metagenomic DNA was performed from 2 g of the rhizosphere soil sample using FastDNA™ SPIN Kit (MPBio, USA) following manufacturer’s instructions. The content, purity, and quality of the extracted DNA was determined using NanoDrop 1000 spectrophotometer (NanoDrop Technologies Inc., USA). Extracted metagenomic DNA was checked on agarose-gel electrophoresis. Isolated metagenomic DNA was used for the high-throughput sequencing through Illumina HiSeq Sequencing system. Metagenomic library was constructed and sequence analysis was performed with approximately 2.6 gigabases (Gb) of metagenomic data using bioinformatics tools.

### 2.3. Annotation of Metagenomic Dataset

The paired end fastQ read files of the metagenomic sample was uploaded to the Metagenome Rapid Annotation using Subsystem Technology (MG-RAST) server (http://metagenomics.anl.gov/) [16] and processed through the standard pipeline with default parameters. Sequence similarity searches were done for identification of proteins and other annotations by alignment against different databases, i.e., protein databases M5NR [17], Genbank [18,19], SEED [20] and Kyoto Encyclopedia of Genes and Genomes (KEGG) [21]. All the analysis was performed using 96-node high performance supercomputing system (HP) cluster available at ICAR-National Bureau of Agriculturally Important Microorganisms, Mau, India.

### 2.4. Taxonomic and Functional Annotation

For the taxonomic assignments, sequence alignment was done through the MG-RAST pipeline against the RefSeq protein database which allows accessibility to different sequence databases as a single, searchable database [16]. Parameters applied were maximum E-value of 1 × 10^−5^, a minimum percentage identity of 60%, and a minimum alignment length of 15. For functional analysis comparisons, subsystems were implied to assign functional roles to genes [22,23].

### 2.5. Metabolic Potential Analysis

For functional annotation of different metabolic pathways, Kyoto Encyclopedia of Genes and Genomes (KEGG) was used from the MG-RAST pipeline (parameters: minimum alignment length of 15 and E-value cutoffs of 1e-5). Metabolic genes for different biological processes were identified in the dataset and the abundances of those gene sequences were calculated. 

### 2.6. Availability of Data and Associated Information

The metagenome dataset with information about taxonomic and functional assignments with other details is publicly available from the MG-RAST with ID mgm4776125.3.

## 3. Results and Discussion

Millet crops have gained worldwide attention due to their intrinsic resistance against diseases, nutritional value to feed subsistence rural population, ability to grow under dry and harsh conditions, and environmental robustness. Physicochemical analysis of kodo rhizosphere suggested that the soil was low in carbon (0.49%), nitrogen (available N 195.7 kg/ha),and phosphorus (available P 9.46 kg/ha) content. The soil was almost normal with pH 7.15 and moderate in potassium (available K 167.2 kg/ha) content. The soil analysis revealed that the kodo millet plants were growing well in low carbon and nutrient deficient soils in the water deficient conditions. 

### 3.1. Sequencing and Annotation of Proteins

A total of 2.6 GB data was obtained for kodo rhizosphere metagenome with 10,669,925 sequences. Out of the total sequences, 590,010 (5.53%) failed to pass the QC pipeline. Furthermore, 516,927 sequences were identified as artificial duplicate reads. Of the sequences that passed quality control, certain sequences (11,748) were predicted as ribosomal RNA genes (0.13%) while 33.13% (3,086,627) were identified as predicted proteins with known functions and 66.75% (6,218,676) as predicted proteins with unknown functions.

### 3.2. Taxonomic Microbial Diversity in the Kodo Rhizosphere

**Figure 1:**
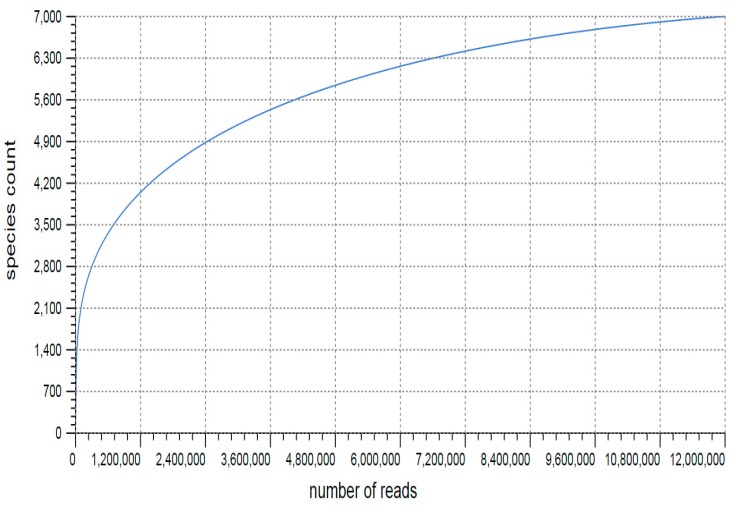
Rarefaction curve of species richness for kodo rhizosphere microbiome.

The taxonomic classification of genes was carried out through Best Hit Classification algorithm of MG-RAST against the M5NR database [16]. A total of 9,317,051 sequences were assigned to various taxonomies. There were also a number of sequences that could not be assigned to the highest taxonomy levels with an average of 15.5% of the reads and they were tagged as either unassigned, unclassified, or ‘others’. Over all, bacteria dominantly accounted for 98.12% of the total assigned reads followed by Eukaryota (1.21%) and Archaea (0.58%), while ‘unclassified sequences’ and ‘other sequences’ accounted only for 0.06 and 0.01% respectively. Viral sequences were least abundant in the rhizosphere sample with only 0.01% fraction. The alpha diversity for the kodo rhizobiome was calculated to be 381 reflecting the presence of significant number of species. The rarefaction curve predicted the taxonomic diversity and reflected significantly high level of community variability in the kodo rhizosphere (Figure 1).

### 3.3. Community Composition and Abundance

**Figure 2:**
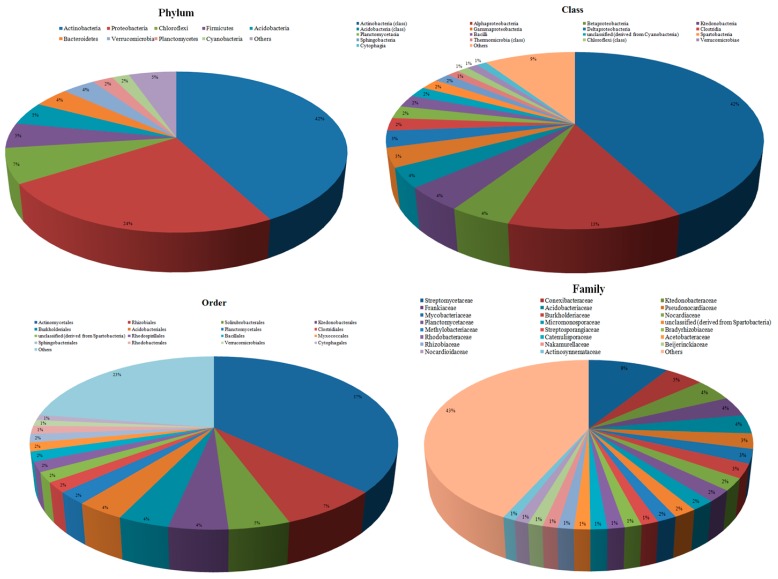
The dominance of different microbial communities in the kodo rhizobiome at different taxonomic units. Groups with less than 1% reads were clubbed together and designated as ‘others’.

A total of 65 different phyla were identified for the reads along with those characterized as ‘unclassified sequences’ (derived from Archaea, Bacteria, Eukaryota, Fungi, other sequences, unclassified sequences or viruses). Among all the phyla, Actinobacteria (42.22%) and Proteobacteria (23.72%) were the most dominating communities with almost 66% of total reads. Other dominant bacterial phyla included Chloroflexi (7%), Firmicutes (5.08%), Acidobacteria (4.65%), Bacteroidetes (4%), Verrucomicrobia (3.7%), Planctomycetes (2.32%), and Cyanobacteria (1.95%). Rest of the phyla possess less than 1% of total reads (Figure 2).

At class level, 205 different classes were identified. Actinobacteria was the most dominant class occupying 42.22% of the total assigned reads followed by Alphaproteobacteria (12.57%), Betaproteobacteria (4.45%), Ktedonobacteria (4.42%), Acidobacteria (3.61%), Gammaproteobacteria (3.48%), Deltaproteobacteria (3.05%), Clostridia (2.33%), Planctomycetacia (2.32%), Bacilli (2.29%), unclassified (derived from Cyanobacteria) (1.92%), Spartobacteria (1.90%), Sphingobacteria (1.6%), Thermomicrobia (1.23%), Chloroflexi (1.18%), Verrucomicrobiae (1.12%), and Cytophagia (1.02%). Less than 1% of the entire reads was individually assigned to rest of the classes which cumulatively accounts for 9% only (Figure 2). 

At the order level, hits were obtained for 554 different taxa with maximum hits for Actinomycetales (36.87%). Only 16 orders occupied more than 1% of the reads though rest of the orders cumulatively accounted for 22.54% of the total distribution (Figure 2). Family-level taxonomy indicated 1073 identified families. However, only 23 families were with more than 1% of the total read distribution. Most of the dominantly present families were observed for the order Actinomycetales (26.03%) including Streptomycetaceae (9.45%) and 10others (Frankiaceae: 3.80%, Pseudonocardiaceae: 2.91%, Mycobacteriaceae: 2.70%, Nocardiaceae: 2.33%, Micromonosporaceae: 1.96%, Streptosporangiaceae: 1.43%, Catenulisporaceae: 1.37%, Nakamurellaceae: 1.24%, Nocardioidaceae: 1.2%, and Actinosynnemataceae: 1.05%). We noticed that 42.78% of the total reads were assigned to rest of 1050 families with less than 1% of reads. 

Similarly, at genus level reads were assigned to 2115 different genera with only 17 genera having more than 1% reads. Rest of the genera cumulatively accounted for 55.75% distribution (Figure 2). In these 17 genera, Streptomyces (9.37%) was the most dominant community while nine other genera also belonged to the order Actinomycetales (Frankia, Mycobacterium, Rhodococcus, Micromonospora, Streptosporangium, Catenulispora, Amycolatopsis, Nakamurella, and Actinosynnema). 

Actinomycetales is the taxonomic order of Actinobacteria with largest taxonomic units amongst 18 identified lineages within the bacteria [24,25]. The members are referred to Actinomycetes, the species of which are known for producing prominent antimicrobial compounds—such as streptomycin, actinomycin, and streptothricin of immense agricultural importance [26,27]. The order Streptomycetales, especially the genus Streptomyces synthesizes almost 80% of the total metabolites known today as compared to other unicellular bacteria except *Bacillus* and *Pseudomonas* species (16%), cyanobacteria (3.7%), and myxobacteria (1.8%) [28]. These communities serve as valuable sources of novel secondary metabolites with multiple biological functions such as antagonism, anti-infection, anticancer, and antibiotics [29,30,31,32]. The dominance of Actinobacteria in the kodo rhizosphere reflects significant attributions with respect to the crop robustness against diseases, and that too under stressed environment. These communities have a proven role to protect plants under oxidative stress conditions [32,33], their dominance indicates their supportive functions for the kodo crop being grown in dry environment. Apart from this, the revelations on the dominance of Streptomycetales community in the kodo rhizosphere may invite attention for the isolation and identification of antibiotic-producing cultivable actinobacteria using culturable strategies. 

The second most dominating family and genus of Actinomycetales was *Frankia*, a N-fixing actinomycete forming root nodules [34]. These communities fix nitrogen under free-living and symbiotic conditions [25,35,36]. Since kodo is grown in fertility-deprived soil and the crop requirement for N as external input is low (40 kg N per ha) (http://vikaspedia.in/agriculture/crop-production/package-of-practices/cereals-and-millets/finger-millet-and-kodo-millet; website visited on 17.9.2019), the dominance of N-fixer *Frankia* species in the rhizosphere reflects prominent role of these communities in supporting N-demand of the plants under low nitrogen conditions. 

### 3.4. Metabolic Multifunctionalities in the Kodo Rhizosphere

**Figure 3:**
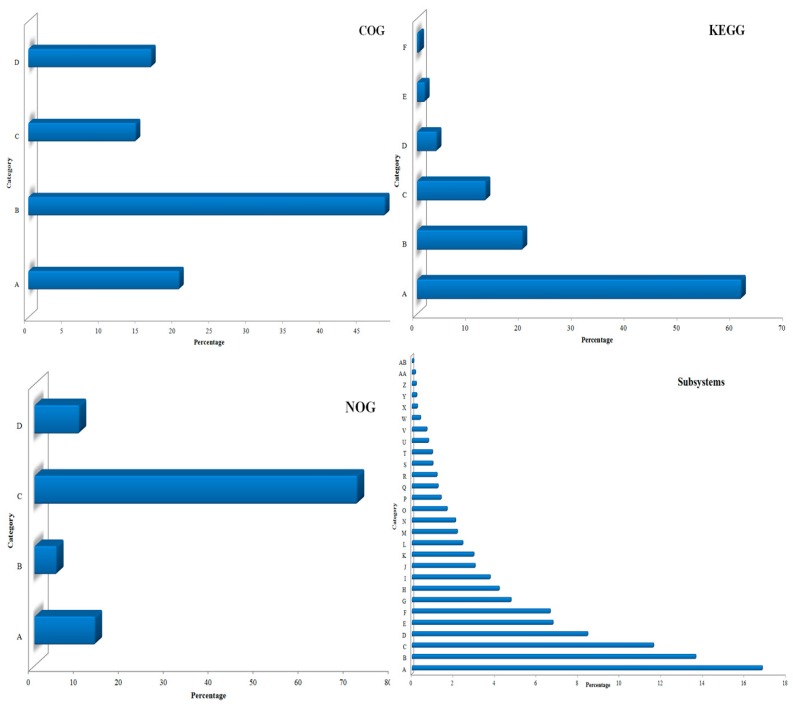
Distribution of reads to different functional categories using COG, KEGG, NOG, and Subsystems databases. Abbreviations used are, **COG** (A: Information Storage And Processing, B: Metabolism, C: Poorly Characterized, D: Cellular Processes And Signaling); **KEGG** (A: Metabolism, B: Genetic Information Processing, C: Environmental Information Processing, D: Cellular Processes, E: Human Diseases, F: Organismal Systems), **NOG** (A: Information Storage and Processing, B: Cellular Processes and Signaling, C: Poorly Characterized, D: Metabolism); **Subsystems** (A: Carbohydrates, B: Clustering-based subsystems, C: Amino Acids and Derivatives, D: Protein Metabolism, E: Miscellaneous, F: Cofactors, Vitamins, Prosthetic Groups, Pigments, G: RNA Metabolism, H: DNA Metabolism, I: Fatty Acids, Lipids, and Isoprenoids, J: Cell Wall and Capsule, K: Respiration, L: Nucleosides and Nucleotides, M: Virulence, Disease and Defense, N: Stress Response, O: Membrane Transport, P: Metabolism of Aromatic Compounds, Q: Phages, Prophages, Transposable elements, Plasmids, R: Cell Division and Cell Cycle, S: Regulation and Cell signaling, T: Phosphorus Metabolism, U: Nitrogen Metabolism, V: Sulfur Metabolism, W: Motility and Chemotaxis, X: Secondary Metabolism, Y: Iron acquisition and metabolism, Z: Potassium metabolism, AA: Dormancy and Sporulation, AB: Photosynthesis).

Microbial communities inhabiting rhizosphere soil play crucial biogeochemical role in the root microenvironment [37]. Functional characterization of the rhizobiome thus becomes crucial for the understanding of microbial support to the plants in nutrient-poor, abiotic stressed and disease prone conditions [38,39]. Protein function in the dataset was identified through evidence-based annotations (COG, KEGG, SEED SubSystem) classified into diverse hierarchies i.e. particular genes, protein families and cellular processes. Identified proteins revealed community-linked metabolic potentials and functional activities of the kodo rhizosphere. COGs classification reflected that ‘Metabolism’ (48.33%) was the most abundant functional category followed by ‘Information Storage and Processing’ (20.45%) and ‘Cellular Processes and Signaling’ (16.65%) (Figure 3). ‘Poorly characterized’ (14.57%) category was also noticed. KEGG also showed ‘Metabolism’ (61.37%) as the most abundant category followed by ‘Genetic Information Processing’ (20%), ‘Environmental Information Processing’ (13%), ‘Cellular Processes’ (3.74%), ‘Human Diseases’ (1.5%), and ‘Organismal Systems’ (0.43%) (Figure 3). Hierarchical analysis of Level 1 of SEED Subsystems again reflected different prominent functions of microbial communities in the rhizopshere. Sequences associated with carbohydrate metabolism, amino acids and derivatives, protein metabolism, cofactors, vitamins, prosthetic groups, pigments and RNA and DNA metabolism were abundant (Figure 3). Metaproteomics [40,41] and metatranscriptomics studies [42,43,44,45] have already been reported for the presence of such functional categories in different soil, sediment and water ecosystems. These functions were shown to be linked with the maintenance and regulation of basic cellular processes that support growth and metabolism of microbial communities under various environments [46,47]. We speculate that with the abundance of these functions, the communities harboring kodo rhizosphere maintain and regulate their own cellular functions and metabolism.

### 3.5. Carbon Fixation

The analysis indicated that the genes related to the pathways involved in the central carbohydrate metabolism and energy generation (Entner–Doudoroff pathway, glycolysis and gluconeogenesis, and pentose phosphate pathway) were significantly present in the dataset. Abundant hits were observed for carbon dioxide fixation along with the pathways of Calvin–Benson cycle, carboxysome, CO_2_ uptake, and photorespiration (oxidative C2 cycle). Different level 2 stages, their associated pathways and enzymes related to carbohydrate metabolism including amino sugars (GlcNAc) 2 catabolic operon, chitin and N-acetylglucosamine utilization, N-acetyl-galactosamine and galactosamine utilization, neotrehalosadiamine ((NTD) Biosynthesis Operon); central carbohydrate metabolism (including glycolysis, gluconeogenesis) were identified. In the kodo rhizosphere, the communities that utilize amino sugars, polysaccharides, organic acids, one-carbon compounds, and sugar alcohols as sole source of carbon and energy were observed. The communities that participate in the central carbohydrate metabolism, fermentation, and hydrolysis were also identified for their food and energy related needs. This is eventually helpful for survival of kodo plant as biochemical analysis reflects low organic content of soil. It reflects that rhizosphere microbial communities are helping the plant to meet their demand for carbon through different metabolic pathways as soil exhibits low carbon content.

### 3.6. Mineral Metabolism

The dataset was analyzed for different hierarchies (level 2, level 3, and functions) with the SEED subsystems to explore genes linked with the major functions in nitrogen, phosphorus, sulfur, and iron metabolism

#### 3.6.1. Nitrogen Metabolism

**Figure 4:**
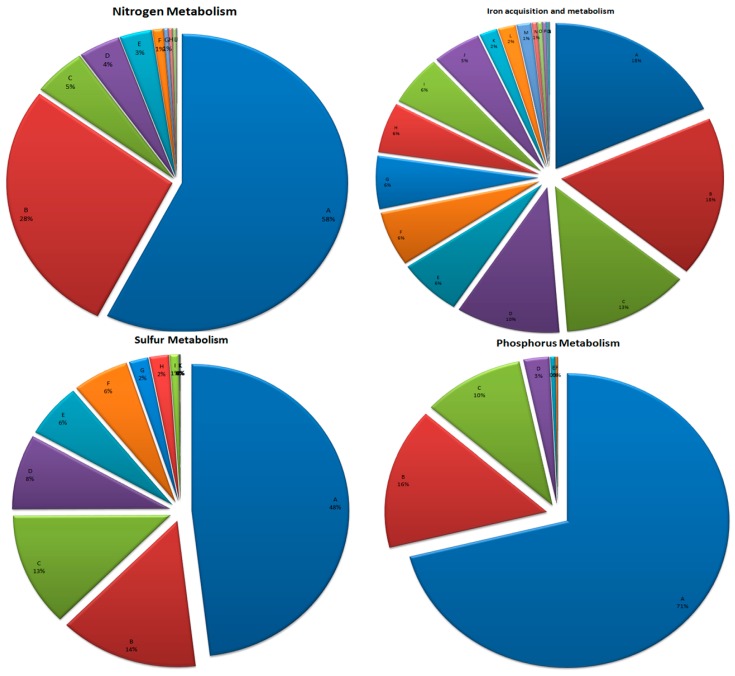
Assignment of reads in different pathways of the different biological process. Percentage distribution of different pathways in a particular biological process is shown. Abbreviations used are, **Nitrogen Metabolism** (A: Ammonia assimilation, B: Nitrate and nitrite ammonification, C: Denitrification, D: Nitric oxide synthase, E: Allantoin Utilization, F: Nitrogen fixation, G: Amidase, H: Cyanate hydrolysis, I: Nitrosative stress, J: Nitrilase, K: Dissimilatory nitrite reductase), **Sulfur Metabolism** (A: Inorganic Sulfur Assimilation, B: Sulfur oxidation, C: Alkanesulfonate assimilation, D: Utilization of glutathione as a sulphur source, E: Thioredoxin-disulfide reductase, F: L-Cystine Uptake and Metabolism, G: Alkanesulfonates Utilization, H: Taurine Utilization, I: Galactosylceramide and Sulfatide metabolism, J: DMSP breakdown, K: Release of Dimethyl Sulfide (DMS) from Dimethylsulfoniopropionate (DMSP), L: Sulfate reduction-associated complexes), **Iron acquisition and metabolism** (A: Campylobacter Iron Metabolism, B: Siderophore Pyoverdine, C: Transport of Iron, D: Iron acquisition in Vibrio, E: Ferrous iron transporter EfeUOB, low-pH-induced, F: Bacillibactin Siderophore, G: Siderophore assembly kit, H: Encapsulating protein for DyP-type peroxidase and ferritin-like protein oligomers, I: Heme, hemin uptake and utilization systems in GramPositives, J: Heme, hemin uptake and utilization systems in GramNegatives, K: Siderophore Yersiniabactin Biosynthesis, L: ABC-type iron transport system, M: ABC transporter (iron.B12.siderophore.hemin), N: Hemin transport system, O: Siderophore pyochelin, P: Iron(III) dicitrate transport system Fec, Q: Iron Scavenging cluster in Thermus, R: Siderophore Achromobactin, S: Siderophore Enterobactin), **Phosphorus Metabolism** (A: Phosphate metabolism, B: High affinity phosphate transporter and control of PHO regulon, C: P uptake (cyanobacteria), D: Alkylphosphonate utilization, E: Phosphonate metabolism, F: Phosphoenolpyruvate phosphomutase, G: Phosphate-binding DING proteins).

Reads related to ammonia assimilation (58%) occupied a maximum of the total sequences of N metabolism followed by ammonification (28%) and denitrification (5%). The proportion of the reads linked with N fixation was low (1% only) (Figure 4). A minor fraction of the reads matched with the processes like allantoin utilization and cyanate hydrolysis. The abundance of reads linked with ammonia assimilation in the plant rhizosphere followed by those associated with nitrate and nitrite ammonification is indicative of such processes that enhance nitrogen use efficiency (NUE) in the plants [48] that grow under low N availability [49]. Since ammonia can be directly assimilated in to amino acids, few pathways like glutamate, alanine, or aspartate and other cellular components are known for its assimilation. Enzymes like glutamate dehydrogenase (GDH), glutamine synthetase (GS), and glutamate synthase are the major catalyzing agents for these reactions. Reads associated with the enzymes glutamate synthase (EC 1.4.1.13), glutamate-ammonia-ligase adenylyltransferase (EC 2.7.7.42), nitrogen regulation protein (NR(I) and NR(II)), nitrogen regulatory protein (P-II), and ammonium transporter indicate prominent assimilation of ammonia in the rhizosphere inhabiting microbial communities. Certain bacteria possess the ability to reduce nitrate and/or nitrite to ammonium (NH_4_^+^) without nitrous oxide as an intermediate. The process, known as nitrate/nitrite ammonification improves NUE because the end product, i.e., ammonia is retained in the soils for utilization by the plants. The reads related to the nitrate/nitrite ammonification (28%) were dominant in the kodo rhizosphere. The enzymes such as nitrate ABC transporter, nitrate/nitrite transporter, nitrite reductase (EC 1.7.1.4), Respiratory nitrate reductase (EC 1.7.99.4), nrfE, NrfC protein, response regulator NasT responsible for these processes were identified in the rhizosphere metagenome. Functionally, the dominance of these processes due to dominant microbial communities with such functionalities may enrich soil N content.

We further explored the reads for different enzymes that help incorporation of nitrogen in the plants [50]. Prominent enzymes allantoate amidohydrolase (EC 3.5.3.9), allantoicase (EC 3.5.3.4), allantoinase (EC 3.5.2.5), ureidoglycolate dehydrogenase (EC 1.1.1.154), and ureidoglycolate hydrolase (EC 3.5.3.19) related to allantoin utilization were identified in the dataset. Sequences linked with the methylobacterium, which is known for allantoin utilization [51] are present significantly (Figure 4). Presence of such communities and their processes are therefore, indicative of improving N economy in rhizosphere through enhanced NUE under nitrogen limitations. Rhizosphere soil analysis has indicated deficiency of N, which seems to be compensated by the presence of N fixing and assimilating microbial communities. Thus, the microbial communities are helping the plants to cope up with less N content through making environmental nitrogen available.

The presence of different cyanate ABC transporter proteins, cyanate hydratase (EC 4.2.1.104) and Cyn operon transcriptional activator involved in cynate hydrolysis was traced. Cyanate is generally formed spontaneously inside the cells from urea and carbamoylphosphate [52,53] or appear in the environment as a result of physicochemical decomposition of urea or cyanide [54,55]. Certain marine cyanobacteria [56,57] and only one identified organism, *Nitrososphaera gargensis* [58] utilize cyanate as N source for energy under N-limiting conditions [59]. The presence of the reads linked with the cyanate metabolism in kodo rhizosphere may be an interesting finding. 

Different enzymes including nitrite reductase (EC 1.7.2.1), nitric-oxide reductase (EC 1.7.99.7), nitrous-oxide reductase (EC 1.7.99.6), transcriptional regulator and maturation protein for denitrification were identified. Though denitrification is a process of losing N from the soils, the presence of enzymes with denitrifying activities, even in low proportion (5%) reflects a naturally balanced ecosystem, where microbial communities normally return fixed N to the atmosphere [60]. For N fixation, reads linked to homocitrate synthase (EC 2.3.3.14), nitrogenase, *NifA* and *VnfA* were identified. Certain reads were also identified for the enzymes involved in the processes of N metabolism like nitrosative stress (NorR, NnrS), dissimilatory nitrite reductase (Cytochrome c551 NirM), nitric oxide synthase (putative cytochrome P450 hydroxylase), and nitrilase (Plant-induced nitrilase; EC 3.5.5.1). Altogether the results generate an insight that in the kodo rhizosphere, nitrogen economy is mainly maintained by the processes like ammonia assimilation, nitrate/nitrite ammonification, allantoin utilization, and N fixation (Figure 4). The soils in which these plants grow are usually N deficient and the N fertilizer usage in kodo crop is very limited [49]. In the rhizosphere, plants, microbial communities, and other soil inhabitants interdependently depend on the naturally managed low N resources for their nitrogen requirements. This has major implications for kodo plants because N is a crucial element for the growth and development.

#### 3.6.2. Phosphorus Metabolism

Phosphorus (P) is an essential macronutrient. It also manages low pH stress through cytoplasmic buffering of hydrogen ions [61]. Limited availability of P in the soils, freshwater, and marine ecosystems influences primary and heterotrophic bacterial productivity [62]. The rhizosphere of the kodo is low in P as is evident from the soil analysis. Microorganisms acquire inorganic and organic forms of reduced P compounds like phosphonate, phosphite, and hypophosphite [63]. The most abundant gene pool (71%) for P metabolism in the dataset included different Phn proteins, phosphate metabolism (different phosphatase, NAD(P), transhydrogenase and various enzymes (EC 3.1.3.1, EC 3.6.1.11, EC 3.6.1.1, EC 1.6.1.2, EC 2.7.4.1, EC 3.6.1.1, EC 1.6.1.1), phosphoenolpyruvate phosphomutase, phosphonate metabolism (different ABC transporter) proteins along with phosphate-binding DING proteins (Figure 4). Different enzymes prominently related to high-affinity phosphate transporter and control of PHO regulon (16%), P-uptake (10%), and alkylphosphonate utilization were indicative of enhanced plant P availability, P-solubilization, and mineralization through the acquisition of P from phosphonates with the help of rhizosphere microbial communities, as is evident from earlier studies [64]. 

#### 3.6.3. Sulfur Metabolism

In the kodo rhizosphere, 48% genes were linked with inorganic sulfur assimilation and those involved in galactosylceramide, sulfatide metabolism, and sulfur oxidation. Enzymes known for inorganic sulfur assimilationlike ABC-type probable sulfate transporters, adenylylsulfate reductase, ferredoxin, oxidoreductase, sulfate adenylyltransferase, sulfate transport system, and permease proteins were identified. Genes related to galactosylceramide and sulfatide metabolism, sulfate reduction-associated complexes, sulfur oxidation, and thioredoxin-disulfide reductase were also detected. For organic sulfur assimilation, genes and pathways related to alkanesulfonate assimilation, DMSP breakdown, L-Cystine uptake/metabolism, and utilization of taurine and glutathione as a sulfur source were identified (Figure 4). Of the two sulfur oxidation pathways, the Sox is involved in complete oxidation of reduced sulfur compounds to sulphate while the APS involves adenosine-5-phosphosulphate as an intermediate [65,66]. A completely functional Sox complex includes SoxB (key component), SoxXA, SoxYZ, and SoxCD components [64,65]. Reads linked with sulfur oxidation (14%) showed SoxB along with SoxA, SoxX, and SoxY suggesting the occurrence of sulfur oxidation processes in the rhizosphere. Sulfur-oxidizing bacterial communities include members from Alpha-, Beta-, Gamma- and Epsilon-proteobacteria, Chlorobia and Chloroflexi along with the photo- and chemoautotrophic bacteria [65,66] while the sulfur-reducing bacteria are mostly from Deltaproteobacteria [66,67,68,69]. Interestingly, a wide assemblage of these communities inhabited kodo rhizosphere as is evident from their taxonomic abundance (Figure 2). Apart from inorganic sulfur assimilation and sulfur oxidation, reads linked with alkanesulphonate assimilation (13%), utilization of glutathione as sulfur source (8%), thioredoxin-disulfide reductase, and L-cystine uptake and metabolism (both 6%) were also present(Figure 4). The role of the enzymes associated with sulfur metabolism have been reported from microbial communities inhabiting various habitats [70,71,72,73]. Their presence in metagenome reflects a balanced sulfur metabolic capability of microbial communities associated with the kodo rhizosphere. 

#### 3.6.4. Iron Acquisition and Metabolism

Iron (Fe) is a crucial micronutrient for the living organisms for activating metabolic enzymes and pathways as prosthetic group constituent [74]. High-affinity Fe transport systems involving biosynthetic chelates, the siderophores help microorganisms and plants to tolerate Fe stress. Transport systems allow microorganisms to competitively obtain Fe as siderophores, to which plants utilize under varied soil conditions. We identified gene sequences related to iron acquisition and metabolism in the kodo rhizosphere metagenome through SEED subsystem alignment at different levels (Figure 4). Sub-categorization of these sequences further revealed that most of them were associated with siderophore activity plus some other functions, e.g. ABC transporter, heme, hemin uptake and utilization systems (gram negative and gram positive both), hemin transport system, Iron(III) dicitrate transport system, iron acquisition in *Vibrio*, iron scavenging cluster in *Thermus*, iron metabolism in *Campylobacter*, and iron transport (Figure 4). A large number of siderophore related sequences were involved in the siderophore assembly i.e. ABC-type Fe^3+^-siderophore transport system, Ferric hydroxamate ABC transporter (EC 3.A.1.14.3), Isochorismate synthase (EC 5.4.4.2) of siderophore biosynthesis, Siderophore biosynthesis protein, Siderophore synthetase component and TonB-dependent proteins. Different siderophores linked gene sequences that resembled pyoverdine (generally produced by the members of the family Pseudomonaceae, i.e., *Azotobacter*, *Azomonas*, *Pseudomonas*, and *Rhizobacter*) [75] , achromobactin (siderophore produced by *Pseudomonas syringe*) [76]; yersiniabactin (siderophore of the pathogenic bacteria *Yersinia pestis*, *Yersinia pseudotuberculosis*, and *Yersinina enterocolitica*) [77]; bacillibactin (siderophore synthesized by the genus *Bacillus*) [78]; enterobactin and pyochelin (siderophore synthesized by *Pseudomonas aeruginosa*) [79,80] were traced in the dataset. We speculate that the dominant presence of microbial communities linked with the efficient iron acquisition functions is supposed to make them enable to enhance iron bioavailability in the rhizosphere of kodo plants.

### 3.7. Metabolism of Aromatic Compounds

**Figure 5:**
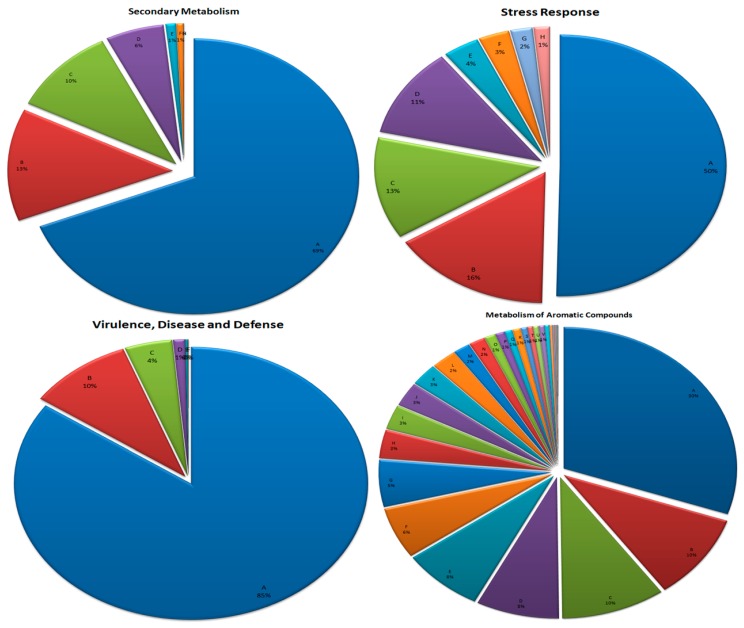
Assignment of reads in different pathways of the different biological process. Percentage distribution of different pathways in a particular biological process is shown. Abbreviations used are, **Secondary Metabolism** (A: Plant hormones, B: Bacterial cytostatics, differentiation factors and antibiotics, C: Plant alkaloids, D: Biosynthesis of phenylpropanoids, E: Aromatic amino acids and derivatives, F: Biologically active compounds in metazoan cell defense and differentiation, G: Plant octadecanoids), **Virulence, Disease and Defense** (A: Resistance to antibiotics and toxic compounds, B: Other, C: Bacteriocins, D: Adhesion, E: Toxins and super-antigens, F: Invasion and intracellular resistance), **Stress Response** (A: Oxidative stress, B: Osmotic stress, C: Heat shock, D: Others, E: Detoxification, F: Acid stress, G: Cold shock, H: Periplasmic stress), **Metabolism of Aromatic Compounds** (A: n-Phenylalkanoic acid degradation, B: Phenylacetyl-CoA catabolic pathway (core), C: Anaerobic benzoate metabolism, D: Benzoate transport and degradation cluster, E: Homogentisate pathway of aromatic compound degradation, F: Catechol branch of beta-ketoadipate pathway, G: Protocatechuate branch of beta-ketoadipate pathway, H: Phenylpropanoid compound degradation, I: Central meta-cleavage pathway of aromatic compound degradation, J: 4-Hydroxyphenylacetic acid catabolic pathway, K: Benzoate degradation, L: Gentisare degradation, M: p-Hydroxybenzoate degradation, N: Biphenyl degradation, O: Chloroaromatic degradation pathway, P: N-heterocyclic aromatic compound_degradation, Q: Acetophenone carboxylase 1, R: Salicylate and gentisate catabolism, S: Carbazol degradation cluster, T: Chlorobenzoate degradation, U: Naphtalene and antracene degradation, V: Aromatic amin catabolism).

Microbial capabilities for utilization of aromatic compound through degradation of xenobiotic chemicals is essential for detoxification of natural habitats [81,82]. We identified sequence reads linked with different pathways involved in the anaerobic degradation of aromatic and xenobiotic compounds, metabolism of aromatic intermediates, and peripheral pathways for catabolism of aromatic molecules (Figure 5). Identified gene sequences were linked with anaerobic toluene and ethylbenzene degradation; 4-hydroxyphenylacetic acid catabolic pathway; catechol and protocatechuate branch of beta-ketoadipate pathway; central meta-cleavage of aromatic compounds; homogentisate and N-heterocyclic degradation pathways; pathways for degradation of benzoate, gentisare, biphenyl, chloroaromatic, chlorobenzoate, naphthalene, and antracene; n-phenylalkanoic acid, phenylpropanoid compound, p-hydroxybenzoate, quinate, salicylate ester, and toluene (Figure 5).

Traces of anaerobic benzoate metabolism, a key intermediary in the microbial metabolism of aromatic compounds [37,83] were detected in the dataset. We identified genes catalyzing aromatic amine catabolism, a constituent of herbicide degradation and causal factor behind bladder cancer [84]. We also detected genes related to enzymes for catabolism of salicylate and gentisate, recognized intermediates in the naphthalene catabolism [85,86] in the rhizosphere microbiome. Carbazole is among the most abundant nitrogeneous compounds from petroleum [87,88]. Likewise, Phenylacetyl-CoA is the component of various substrates like phenylalanine, lignin-related phenylpropane units, phenylalkanoic acids and environmental contaminants such as styrene and ethylbenzene [89]. In the kodo metagenome, genes related to the enzymes associated with phenylacetyl-CoA catabolic pathway (core) were also identified. Collectively, the results confirm that the microbial communities in the rhizosphere of kodo plants with xenobiotic degradation capabilities hold immense promise for bioremediation of soils from the aromatic compounds. 

### 3.8. Secondary Metabolism

Secondary metabolism is one of the most diverse features of microbial communities [90,91]. A wide range of small molecule metabolites are synthesized by microorganisms as a representation of metabolic complexity [92,93]. Being essential tool for self-defense, they play major role in host–microbe, microbe–microbe, and microbe–environment interactions [94] and act as clinically-used antibiotics, antimicrobials, anticancer agents, immuno-suppressants, and other drugs [93,95]. Besides other microorganisms, Streptomycetales are functional Actinobacterial communities to produce diverse secondary metabolites, especially polyketide and peptide-type antibiotics [96,97,98].

Biological processes and pathways that were prominently identified belonged to the aromatic amino acids and derivatives (cinnamic acid degradation, pyrrolnitrin biosynthesis), bacterial cytostatics, differentiation factors, antibiotics (2-isocapryloyl-3R-hydroxymethyl-gamma-butyrolactone) and bacterial morphogens, clavulanic acid biosynthesis, nonribosomal peptide synthetases (NRPS) in *Frankia* sp. Ccl3, paerucumarin biosynthesis, phenazine biosynthesis, biologically active compounds in metazoan cell defense and differentiation (quinolinic acid and its derivatives, steroid sulfates), biosynthesis of phenylpropanoids (flavanone, phytoalexin, phytosterol, salicylic acid and tannin biosynthesis, phenylpropionate degradation), lipid-derived mediators (cannabinoid biosynthesis), alkaloids biosynthesis from l-lysine, phytohormones (auxin biosynthesis, auxin degradation), and octadecanoids (Figure 5). This information is helpful in investigating the rhizobiome for specific microorganisms through improved culturable methods for obtaining efficient strains with potential secondary metabolic functions. Immense benefits from the belowground microbial dark matter (hidden communities, unexplored metabolites) can also be obtained in terms of novel genes and metabolic pathway machinery with diverse chemistry [99,100].

Genes related to enzymes involved in the auxin biosynthesis (aromatic-L-amino-acid decarboxylase (EC 4.1.1.28), indole-3-pyruvate decarboxylase (EC 4.1.1.74), nitrilase 1 (EC 3.5.5.1) and 2 (EC 3.5.5.1), phosphoribosylanthranilate isomerase (EC 5.3.1.24), tryptophan synthase alpha chain (EC 4.2.1.20), tryptophan synthase beta chain (EC 4.2.1.20)) were identified. Growth regulators like cytokinins and auxin (indole-3-acetic acid; IAA) are microbial products affecting the cell division and elongation in plants [101,102]. Such findings reflected that kodo rhizobiome is rich in the auxin producing and secreting microbial communities. Cinnamic acid is a known allelochemical phenolic that influences seed germination, plant root growth, and affects metabolic processes [103]. We identified genes related to enzymes (such as 2,3-dihydroxyphenylpropionate 1,2-dioxygenase, 2-keto-4-pentenoate hydratase, 3-(3-hydroxy-phenyl) propionate hydroxylase, 3-phenylpropionate dioxygenase ferredoxin-NAD(+) reductase component, Hca operon (3-phenylpropionic acid catabolism) transcriptional activator HcaR and Probable 3-phenylpropionic acid transporter) concerned with cinnamic acid biosynthesis. . It is reasonable to speculate that the rhizobiome of this crop has functionalities to downstream the influence of cinnamic acid, and thereby, promotes plant growth and development.

### 3.9. Stress Response

Among the gene sequences identified for stress response, most were related to oxidative stress followed by osmotic and heat stress with an overrepresentation of glycine betaine, glucans, glutathione, and superoxide dismutase (Figure 5). Possible reason for this might be the fact that the crop usually grows in harsh drought stress and nutrient depleted conditions [49] and so, their root associated microbiota are metabolically tuned to such conditions. Apart from the gene sequences related to the enzymes of oxidative, osmotic and heat stress, those involved in acid stress, cold shock, desiccation stress, detoxification, and periplasmic stress were also identified. For oxidative stress, enzymes of various pathways like CoA disulfide thiol-disulfide redox system, glutaredoxins, glutathione: biosynthesis and gamma-glutamyl cycle, glutathione (non-redox reactions and redox cycle both), glutathione analogs: mycothiol, glutathionylspermidine and trypanothione, NADPH:quinone oxidoreductase 2, oxidative stress (general), protection from reactive oxygen species, redox-dependent regulation of nucleus processes, regulation of oxidative stress response were detected (Figure 5). We observed that diverse stress resistance, tolerance and/or avoidance mechanisms are engaged by the microbial communities in the kodo rhizosphere for their survival and performance in the environment. 

Glutathione plays a significant defensive role against oxidative stress [104,105] and provides protection against toxic xenobiotics including environmental pollutants [106,107,108]. We identified genes related to the enzymes of the pathways involved in betaine biosynthesis from glycine, choline, and betaine uptake (also traced multiple copies of betaine biosynthesis sequences), ectoine biosynthesis and regulation, osmoprotectant ABC transporter, osmoregulation, and synthesis of osmoregulated periplasmic glucans. Glycine betaine (GB) is a major organic osmolyte in organisms against different environmental stresses like drought, salinity, high temperature, UV radiation, and heavy metals [109]. Conclusively, the availability of genes related to stress response processes indicates how the inhabiting microbial communities adopt and respond in the rhizosphere microclimate at both the community and organism level and exhibit metabolic capabilities to support growth and development of kodo plants.

### 3.10. Virulence, Disease, and Defense

Significant number of gene sequences linked with virulence, disease, and defense were also identified in the rhizosphere (Figure 5). Such genes include Adhesion (related to *Staphylococcus, Campylobacter*, *Enterobacteria*, and *Streptococcus*), Bacteriocins (bacitracin stress response, marinocine, and tolerance to colicin E2), invasion and intracellular resistance, resistance to antibiotics and toxic compounds (multidrug efflux systems and resistance to arsenic, cadmium, mercury, chromium, zinc, vancomycin, cobalt-zinc-cadmium), toxins and superantigens (diphtheria toxin, streptolysin biosynthesis, and transport). It has been observed that the bacterial communities in the rhizosphere defend themselves through bacteriocins or ribosomally synthesized antibacterial peptides [110]. Adherence is a crucial step of bacterial pathogenesis or infection and colonization with the host [111]. Bacterial adhesins are surface recognition molecules that allow bacteria to target specific surfaces like root tissues [110,111]. The presence of such genes in the rhizosphere pointed out specialized functions of strengthening rhizosphere colonization by inhabiting bacterial communities. Gene sequences related to resistance against metal contamination in the rhizosphere possibly play significant role in bioremediation [62,109]. Metagenomic analysis of different environments including drinking water [112], sediment [113], and soil [114] has led to identify diversity and abundance of antibiotic resistance genes [115,116]. Our analysis, in concurrence with the previous studies, also showed that the microbial communities from kodo rhizobiome have capabilities to bioremediate against metal contamination and defend plants against disease causing organisms. 

Studies suggest that plant associated microbial communities differ widely in the soils and particular plant eventually chooses specific core microbiome [117,118,119] which is supposed to provide key contribution to plant growth and health [120,121,122,123]. Metagenomic analysis of kodo rhizosphere also revealed high taxonomic diversity with actinobacterial dominance (42.22%) along with Proteobacteria (23.72%) and the Bacteroides (4%). The observations are supported by earlier study [124] that on the basis of amplicon metagenome sequencing reflected similar revelations. High throughput sequence analysis of 16S rRNA gene for the assessment of bacterial community composition in three different Andean tuber crops Oca (*Oxalis tuberosa*), Ullucu (*Ullucus tuberosus*), and Mashua (*Tropaeolum tuberosum*) identified Bacteroidetes and Proteobacteria phyla as the most abundant communities [125]. Extensive overlap between core rhizosphere microbiome in different plant species, e.g., citrus [119], *Arabidopsis* [126,127], millet [120], sugarcane [117], and cooloola [128] has been observed to suggest that various factors driving community assembly may become common among plant species. Similarly, analysis of more than 20 wheat rhizosphere metatranscriptomes have led to identify metabolic pathways related to the degradation of aromatic and xenobiotics compounds [129]. Gene sequences linked with these pathways have also been dominantly observed in the kodo rhizosphere.

All together these studies reveal that microbial communities with diverse taxonomic structure and metabolic functions inhabit the crop rhizosphere to contribute in interactive way to support plants in their surrounding environment. Cumulative microbial multifunctionalities in the kodo rhizosphere as is observed in the present study or evidenced from parallel studies on the microbial community structure and function of other crops, ultimately leads to a productive and healthy microenvironment around the plant roots that eventually influences crop survival and productivity. 

## 4. Conclusions

Understanding how different microbial communities in the rhizosphere influence plant performance and productivity using metagenomics opens new avenues for devising eco-friendly ways to cater benefits from microbe-mediated agricultural technologies. Millet crops are in center of attention across the world as they ably grow under nutrient deprived soil conditions, reflect environmental robustness, possess disease resistance and remediation ability, and are high in food nutritional value. 

Accumulating evidences suggest that crop plants in their rhizosphere microenvironment are largely supported by the belowground microbial communities. Metagenomic analysis of kodo rhizosphere revealed high taxonomic diversity with actinobacterial dominance. Further analysis of the metabolic capabilities of microbial communities associated with the kodo rhizosphere has been established with the observations that gene sequences linked with normal physiological pathways, carbon fixation, nutrient cycling and acquisition, stress and defense response, secondary metabolism, xenobiotic degradation, and bioremediation were abundantly identified in the metagenome. With such metabolic functions, the microbial communities are supposed to support growth, development and survival of the crop in the soil under tough environmental conditions. These results established that a rich gene pool is associated with the kodo rhizosphere for: (i) various secondary metabolite pathways associated with synthesis of bacteriocins or ribosomally synthesized antibacterial peptides; (ii) resistance against diverse antimicrobial compounds; and (iii) detoxification of xenobiotic compounds and metals. Knowing these facts, either new culturable strategies can be devised to isolate such microbial strains that can show robust behavior in the adverse condition or model organisms can be manipulated with such genes to harvest novel functional benefits.

The information from mining of metagenome of neglected but agro-ecologically robust and nutritionally sound plants is helpful to identify novel genes and proteins of varied functions. This is also helpful in mapping metabolome of kodo rhizosphere to explore novel small molecules with proven functions of agricultural implications. Conclusively, the availability of genes associated with different important biological processes indicates how the inhabiting microbial communities adopt and respond in the rhizosphere microclimate at both the community and organism level and exhibit metabolic capabilities to support growth and development of kodo plants.
